# Clinical practice guideline on the use of single-operator cholangioscopy in the diagnosis of indeterminate biliary stricture and the treatment of difficult biliary stones

**DOI:** 10.1007/s00464-023-10569-x

**Published:** 2023-12-26

**Authors:** Adriana Margarita Rey Rubiano, Laura Yuriko González-Teshima, Lázaro Arango, Camilo Blanco-Avellaneda, Jhon Jaime Carvajal Gutiérrez, Rodrigo Castaño-Llano, Martin Alonso Gómez Zuleta, Carlos González, Arecio Peñaloza-Ramírez, Raúl Pinilla Morales, Renzo Pinto Carta, Héctor Adolfo Polanía Liscano, Reinaldo Andrés Rincón Sánchez, Mauricio Sepúlveda Copete, Rómulo Vargas-Rubio, Camilo Andrés Avendaño Capriles, Andrés Mauricio García-Sierra, Juan José Yepes-Nuñez

**Affiliations:** 1 School of Medicine, Universidad de los Andes, Carrera 1 # 18a-12, Bogotá, D.C. Colombia; 2 Department of Gastroenterology, Fundación Santa Fe de Bogotá, Bogotá, D.C. Colombia; 3 Department of Clinical Surgical Gastroenterology, University of Caldas, Manizales, Caldas, Colombia; 4 Department of Endoscopy and Gastroenterology, University of Caldas, Manizales, Caldas, Colombia; 5 Union of surgeons, Zentria Group, Manizales, Caldas, Colombia; 6 Department of Gastrointestinal Surgery and Digestive Endoscopy, Pontificia Universidad Javeriana, Bogotá, D.C Colombia; 7 Education and Research Group, Faculty of Education, Universidad El Bosque, Bogotá, D.C. Colombia; 8 Videoendoscopy Unit of Restrepo Ltda, Bogotá, D.C Colombia; 9 Department of Gastroenterology, Hospital Pablo Tobón Uribe, Medellín, Antioquia Colombia; 10 Department of Internal Medicine, Universidad de Antioquia, Medellín, Antioquia Colombia; 11 Department of Gastroenterology, Universidad de Antioquia, Medellín, Antioquia Colombia; 12 Institute of Cancerology, Clínica las Américas Auna, Medellín, Antioquia Colombia; 13 Department of Internal Medicine, Universidad Nacional de Colombia, Bogotá, D.C. Colombia; 14 Department of Internal Medicine, Gastroenterology Section, Hospital Universitario Nacional de Colombia, Bogotá, D.C. Colombia; 15 Department of Gastroenterology and Digestive Endoscopy, Clínica Reina Sofia, Bogotá, D.C. Colombia; 16 Department of Gastroenterology and Digestive Endoscopy, Fundación Universitaria Sanitas, Bogotá, D.C. Colombia; 17 Department of Gastroenterology, Clínica Colombia, Bogotá, D.C. Colombia; 18 Department of Gastroenterology, Fundación Universitaria de Ciencias de la Salud, Bogotá, D.C. Colombia; 19 Department of Gastroenterology, Hospital de San José, Bogotá, D.C. Colombia; 20 Department of Gastroenterology and Oncological Digestive Endoscopy, Instituto Nacional de Cancerología, Bogotá, D.C. Colombia; 21 School of Medicine, Universidad Nacional de Colombia, Bogotá, D.C. Colombia; 22 Colombian Association of Digestive Endoscopy, Bogotá, D.C. Colombia; 23 American Society for Gastrointestinal Endoscopy, Rochester, USA; 24 Department of Gastroenterology, Universidad Surcolombiana, Neiva, Huila Colombia; 25 Endotek Ltda, Hospital Universitario Neiva, Neiva, Huila Colombia; 26 Department of Internal Medicine and Gastroenterology, Pontificia Universidad Javeriana - Hospital Universitario San Ignacio, Bogotá, D.C. Colombia; 27 Department of Gastroenterology, Fundación Clínica Shaio, Bogotá, D.C. Colombia; 28 Department of Gastroenterology, Hospital Universitario de San Ignacio, Pontificia Universidad Javeriana, Bogotá, D.C. Colombia; 29 School of Medicine, Universidad del Norte, Barranquilla, Colombia; 30 Division of Thoracic Surgery and Interventional Pulmonology, Beth Israel Deaconess Medical Center, Harvard Medical School, Boston, MA USA; 31 School of Global Health Management and Informatics, University of Central Florida, Orlando, USA; 32 Pulmonology Service, Internal Medicine Section, Fundación Santa Fe de Bogotá University Hospital, Bogotá, D.C. Colombia; 33 Colombia GRADE Network, Bogotá, D.C. Colombia

**Keywords:** Bile duct diseases, Biliary tract surgical procedures, Clinical practice guideline, GRADE approach

## Abstract

**Background and aims:**

Single-operator cholangioscopy (SOC) offer a diagnostic and therapeutic alternative with an improved optical resolution over conventional techniques; however, there are no standardized clinical practice guidelines for this technology. This evidence-based guideline from the Colombian Association of Digestive Endoscopy (ACED) intends to support patients, clinicians, and others in decisions about using in adults the SOC compared to endoscopic retrograde cholangiopancreatography (ERCP), to diagnose indeterminate biliary stricture and to manage difficult biliary stones.

**Methods:**

ACED created a multidisciplinary guideline panel balanced to minimize potential bias from conflicts of interest. Universidad de los Andes and the Colombia Grading of Recommendations Assessment, Development and Evaluation (GRADE) Network supported the guideline-development process, updating and performing systematic evidence reviews. The panel prioritized clinical questions and outcomes according to their importance for clinicians and patients. The GRADE approach was used, including GRADE Evidence-to-Decision frameworks.

**Results:**

The panel agreed on one recommendation for adult patients with indeterminate biliary strictures and one for adult patients with difficult biliary stones when comparing SOC versus ERCP.

**Conclusion:**

For adult patients with indeterminate biliary strictures, the panel made a conditional recommendation for SOC with stricture pattern characterization over ERCP with brushing and/or biopsy for sensitivity, specificity, and procedure success rate outcomes. For the adult patients with difficult biliary stones the panel made conditional recommendation for SOC over ERCP with large-balloon dilation of papilla. Additional research is required on economic estimations of SOC and knowledge translation evaluations to implement SOC intervention in local contexts.

**Supplementary Information:**

The online version contains supplementary material available at 10.1007/s00464-023-10569-x.

Biliopancreatic diseases are a common occurrence in clinical practice. Although endoscopic retrograde cholangiopancreatography (ERCP) and magnetic resonance imaging (MRI) have been useful tools in the study of these pathologies, they are insufficient in certain cases [1]. Choledocholithiasis has a worldwide prevalence between 5 and 20%, and ERCP with sphincterotomy is the preferred therapeutic strategy, resolving 85–95% of cases [2]. However, in the remaining 5–15% of unsuccessful cases, difficult-to-extract biliary stones occur, and alternative therapies such as large-balloon papillary dilation, mechanical lithotripsy, extracorporeal lithotripsy, and cholangioscopy-guided intraductal laser or electrohydraulic treatment are required for its proper management [2]. Like difficult biliary stones, the diagnosis of the etiology of biliary strictures, which is crucial for the determination of the prognosis of the patients, through ERCP with biopsy and/or brushing, has a poor diagnostic sensitivity (35 to 70%), providing a real challenge for their practitioner [3–5].

Single-operator cholangioscopy (SOC) is a visualization and interventional system used for the diagnostic and therapeutic management of indeterminate strictures and large stones of the biliary system when ERCP is unsuccessful or considered inappropriate [6]. Unlike other techniques, SOC is designed to visualize and facilitate access to the bile ducts during diagnostic and therapeutic procedures, increasing diagnostic sensitivity and specificity up to that can 50–100% and 87–100%, respectively [4–7]. The International Hepato-Pancreato-Biliary Association consensus stated that direct peroral cholangioscopy provides the largest accessory channel, better image definition, and is technically more demanding than conventional methods [8]. Although percutaneous transhepatic cholangioscopy is not new since it was described in 1974 [9], new percutaneous cholangioscopy devices like Spyglass Discover allows even further versatility to the SOC for the management of intrahepatic stones and evaluation of the more proximal strictures of the biliary tree [10, 11]. Even though there are no controlled studies there are multiple case reports supporting the advantages of this new approach that requires a multidisciplinary integration between radiology and endoscopy groups [10, 12, 13]. Another very interesting application of this new device is its usefulness as intraoperative cholangioscopy, which in some cases allows simultaneous management of cholelithiasis and choledocholithiasis [14].

Cholangioscopy is complementary to abdominal imaging and ERCP tissue acquisition in evaluating and diagnosing indeterminate biliary strictures [8]. Therefore, this technique is a promising and logical step to increase diagnostic certainty, despite its high cost [1, 2]. In 2015, the National Institute for Health and Care Excellence (NICE) [6] published a health technology assessment of the SOC-SpyGlass system for the diagnostic and therapeutic management of biliary system diseases, suggesting that this technology should be used when standard techniques are unsuccessful or inappropriate [6]. In 2019, the European Society of Gastrointestinal Endoscopy (ESGE) published a guideline where recommends the use of cholangioscopy-assisted intraluminal lithotripsy (electrohydraulic or laser) as an effective and safe treatment of difficult bile duct stones [15]. However, currently, there are no standardized clinical practice guidelines (CPG) for the use of SOC in diagnosing indeterminate biliary strictures or managing difficult biliary stones at a country level, specifically in the Latin-American region.

This CPG aims to provide evidence-based recommendations on the role of SOC in the diagnosis of indeterminate biliary stenosis and the treatment of difficult-to-manage biliary stones in the Colombian context. This guideline is not intended to be constructed as a standard of care but rather as an aid for clinical decision-making for health workers, based on evidence and in the context of the performance of each clinician and patient.

## Methods

The guideline panel developed and graded the recommendations and assessed the certainty of the evidence following the GRADE framework [16, 17]. The Colombian Association of Digestive Endoscopy (ACED from the Spanish initials for Asociación Colombiana de Endoscopia Digestiva) commissioned to Universidad de los Andes and the Colombia GRADE Network the general development of the guideline, which was derived from the Guidelines International Network–McMaster Guideline Development Checklist (http://cebgrade.mcmaster.ca/guidecheck.html). The Universidad de los Andes and the Colombian GRADE Network assisted in the process of developing the guideline by choosing the GRADE methodology, creating meeting agendas and materials, and moderating panel discussions.

### Organization, panel composition, planning, and coordination

The panel consisted of 14 gastroenterology specialists, of whom 8 had a background in internal medicine and the remaining 6 were trained as surgeons. On average, they had 17.14 (SD ± 6.09) years of experience in conducting endoscopy, and 12 of the panelists had an average of 16.83 years (SD ± 6.04) of expertise in performing ERCP and cholangioscopy procedures. A content expert served as the panel's chair (AMRR). A methodologist with specific knowledge in developing guidelines participated as vice chair (JJYN). Conflicts of interest of all participants were managed based on recommendations of the Institute of Medicine (IOM) [18] and the Guidelines International Network (GIN) [19].

### Guideline funding and management of conflicts of interest

The non-profit organization ACED, which represents gastroenterologists, provided the funding for creating these recommendations. The guidelines panel included members of the ACED. Staff from ACED helped arrange meetings and supported panel selections, but they had not input on selecting the recommendations or the guideline questions. To manage the conflict of interest of all the participants of this CPG, the declaration of conflict of interest of the World Health Organization (WHO) experts was used; this was distributed and compiled using the GRADEpro tool (www.gradepro.org). Only one of the ACED expert panelists reported a conflict of interest due to their business relationship with one of the manufacturers of the cholangioscopy equipment. The rest of the panel manifested not having conflicts of interest. We reported a detailed explanation of the expert panel, the reviewer team, and the conflict-of-interest declaration for each panel member in Supplementary materials 1 and 2. Panel members did not receive additional fees for participating in this guideline. The panelist who declared having a conflict of interest participated in all the deliberation meetings on outcomes and recommendations; however, he abstained from voting to define the recommendations of this guideline.

The researchers associated with Universidad de los Andes and the Colombia GRADE Network in charge of carrying out the systematic literature reviews received a salary according to the agreement between ACED and Universidad de los Andes. None of the researchers associated with Universidad de los Andes and the Colombia GRADE Network declared commercial or financial conflicts of interest associated with developing this guideline.

There was no participation of any group of patients or entities external to those already mentioned.

### Formulating specific clinical questions and determining outcomes of interest

The panel used the GRADEpro Guideline Development Tool (www.gradepro.org) and Microsoft Office 365 Forms [20] to prioritize the clinical questions. Two main questions of interest regarding SOC as a diagnostic tool and its potential use as a therapeutic instrument were established. We conducted a similar process for the outcome prioritization of each question. A consensus definition of each one of the outcomes was made, prioritizing three critical outcomes per question (score > 7 points in the GRADE methodology) [16, 17] (Table 1). Only adult patients (older than 18 years) with biliary strictures of undetermined etiology and difficult biliary stones were considered for diagnosis and treatment questions, respectively. Special interest was placed in those who had undergone SOC with a characterization of the pattern of stricture and biopsy; the latter accompanied by ERCP with brushing and/or biopsy for the case of indeterminate biliary strictures, or by ERCP with large-balloon papillary dilation for the case of difficult-to-extract stones.

**Table 1:** Outcomes prioritized by the panel of experts for decision-making

Prioritized outcome	Score (mean)	Definition
Diagnosis question		
Procedure success rate	8.66	Ability to obtain selective access to the bile duct in one single procedure A procedure that achieves adequate brushing and biopsy in one intervention is considered successful
Diagnostic sensitivity of single-operator cholangioscopy-guided biopsy	8.21	Sensitivity
Diagnostic specificity of single-operator cholangioscopy-guided biopsy	7.71	Specificity
Treatment question		
Time for single-operator cholangioscopy-guided therapeutic maneuvers	7.42	Time of the procedure development in minutes
Number of successful therapeutic procedures	8.14	The procedure that achieves the total extraction of the bile duct stone in a single intervention is considered successful
Incidence of adverse events	7.78	Overall incidence of adverse events

### Evidence reviews and development of recommendations

Using the GRADEpro Guideline Development Tool, the Universidad de los Andes and the Colombia GRADE Network team created a GRADE EtD table for each guideline question [17, 21, 22]. The findings of systematic reviews of the literature that were updated or carried out, especially for these recommendations, were presented in the EtD table. The EtD table addressed the potential benefits and harms of the interventions or the diagnostic test, test accuracy, resource utilization (cost-effectiveness), values and preferences (relative importance of outcomes), equity, acceptability, and feasibility. Before, during, or after the guideline panel meeting, the draft EtD tables were examined by the guideline panel, making suggestions on the adaptability and relevance of the evidence presented to the Colombian context.

The Universidad de los Andes and the Colombia GRADE Network team conducted new systematic reviews of diagnostic tests and intervention strategies following general procedures provided in the Cochrane Handbook for Systematic Reviews (https://training.cochrane.org/handbook). In addition, the reviewer team performed systematic reviews of reviews searching for information about baseline risks (estimation of disease prevalence), values and preferences, equity, acceptability, feasibility, and cost for the Colombian context. We conducted a literature search in two databases: Medline (via Ovid) and Embase. The search was performed up to February 09, 2022, focusing on literature published in English without any other restrictions. (Supplementary material 3).

Using the CADIMA tool (https://www.cadima.info/index.php) the articles were screened by title and abstract, then full text and, finally, data extraction from the screened articles. Panel members also stayed on the lookout for additional suitable studies so they could submit them to the panel for possible inclusion. The risk of bias assessment for diagnostic accuracy studies was performed using the QUADAS-2 tool [23]. For the intervention studies that met the inclusion criteria, we assessed the risk of bias using the Cochrane Risk of Bias Assessment Tool version 1.0 [24]. In the case of observational studies, we used Risk of Bias In Non-randomized Studies—of Interventions (ROBINS-I) tool [25]. We performed statistical analyses of diagnostic test accuracy studies using MetaDisc 2.0 [26] and conducted the metanalysis for the intervention studies with Review Manager 5.4 [27]. For each of the prioritized outcomes, the evaluation of the certainty in the evidence was carried out following the GRADE methodology on the following domains: risk of bias, precision, consistency, magnitude of the estimate of effects, directness of the evidence, and risk of publication bias. The certainty was categorized into 4 levels ranging from very low to high [28–30]. We plan to submit systematic reviews of benefits and harms and test accuracy for future publication. This report follows an outline proposed to report trustworthy guidelines [31].

The panel developed recommendations over two online meetings using the evidence presented in the EtD tables. The panel took a population perspective and agreed on the following for each recommendation: the certainty of the evidence, the healthcare-related desirable and undesirable effects of the compared diagnostic strategy options or interventions, and the assumptions about the values and preferences related to the decision. The panel also agreed on the resource utilization associated with each question's alternative diagnostic options or interventions. Using the GRADE approach, the panel reached a consensus on the direction and strength of recommendations through group discussions and deliberations [32]. This consensus was reached through three online voting sessions, with a quorum of 70% attendance from all members with voting rights during these sessions. The decisions were made based on balancing all desirable and undesirable consequences [17, 21, 22]. Each judgment based on the EtD criteria, and the ultimate recommendation underwent a voting process, with a requirement for a 70% threshold in each vote. The conclusive votes for each evaluated criterion are detailed in the EtD additional considerations section of each question. The final guideline report was reviewed and approved by all panel members. Compliance to AGREE II reporting standards for clinical practice guidelines was evaluated for this guideline (Supplementary material 4).

### Interpretation of strong and conditional recommendations

The recommendations are labeled as either “strong” or “conditional” according to the GRADE approach. The words “the guideline panel recommends” are used for strong recommendations and “the guideline panel suggests” for conditional recommendations. Table 2 provides the interpretation of strong and conditional recommendations by patients, clinicians, and healthcare policymakers.

**Table 2:** Interpretation of strong and conditional recommendations using GRADE

Implications for	STRONG recommendation	CONDITIONAL recommendation
Patients	Most patients would prefer strong recommendations, only a small proportion of patients would not	Most patients would prefer the strong recommendations, however, a sizeable portion of patients would not Support elements that consider the individual risks, values and preferences of patients can facilitate their decision-making process
Healthcare professionals	Most professionals should follow the strong recommendations. No aids are required for the decision-making process based on individual values and preferences	Different options are considered appropriate depending on the individual conditions of each patient. Health professionals must support each patient in their decision-making process according to their values and preferences. Decision aids can be helpful for patients in this process considering their personal risks and values and preferences
Policy makers	The recommendation can be adopted as policy in most situations Adherence to guideline recommendations can be used as an indicator of quality and performance	Converting these recommendations into policy may require multidisciplinary discussion. Performance indicators should measure whether the decision-making process based on the recommendation is adequate
Researchers	The recommendation is supported by reliable evidence and is consistent with the evidence available in the literature to date Sometimes strong recommendations may be based on evidence with a low or very low level of certainty; in these cases, additional information searches can provide crucial data that can alter the recommendations	The recommendation can be strengthened with additional information searches, either updates of the same or adaptations of the available evidence The evaluation of the conditions and criteria (assessments, evidence used, and additional considerations) that marked these recommendations as conditional instead of strong, are the points that will help identify possible gaps in knowledge

### How to use this guideline

The aim of the ACED guideline is to help clinicians make decisions about diagnostic and treatment alternatives. Other objectives include determining future research needs and informing advocacy, instruction, and policy. Patients may also use them. These recommendations are not meant to represent or be taken as a standard of care. Clinicians must decide based on each patient's clinical presentation, ideally through a collaborative approach that considers the patient's values and preferences concerning the selected alternative's expected consequences. The reality of a particular clinical situation and available resources, including but not limited to institutional policies, time constraints, and treatment availability, may place restrictions on decisions. These recommendations may not cover all appropriate treatments for the stated clinical contexts. Recommendations may become outdated as research develops and more data becomes available. Following these recommendations won't guarantee success. No products mentioned in these recommendations are warranted or guaranteed by ACED. Its components, which aid in more accurate interpretation, include statements of the underlying values and preferences and qualifying remarks associated with each recommendation. They should always be included when these suggestions are quoted or translated.

## Results

The key points are presented here; however, the Supplement material provides a comprehensive summary of findings and the detailed EtD framework is shown online, refer to the link specified in each question description. Table 3 shows a summary of the study population.

**Table 3:** Population information from included studies

Study, year	n	Type of study	Description of the population	Country	Intervention	Comparison	Outcomes
Diagnosis of biliary strictures of undetermined etiology							
Gerges, (2020) [7]	57 patients (57 specimens)	Randomized clinical trial	Patients with symptoms of biliary obstruction who were 18 years of age or older and had no contraindications to endoscopy	India, China, Germany	DSOC visualization and sampling for DSOC-guided biopsy	Standard ERCP visualization with tissue brushing	Sensitivity, specificity, procedure success rate
Hartman, (2012) [5]	86 patients (121 specimens)	Retrospective cohort	Patients who had IBS at a single institution. Specimens were obtained from Each specimen consisted of single to multiple biopsy fragments	United States	Spybite forceps guided by cholangioscopy plus direct visualization with Spyglass system	Fluoroscopy-guided ERCP plus standard biopsy with unguided forceps	Sensitivity, specificity, procedure success rate
Management of patients with difficult biliary stones							
Bang, (2020) [37]	66 patients	Randomized clinical trial	Patients with difficult biliary stones recruited from ward or outpatient referral services	United States	SOC-guided laser lithotripsy (Spyglass)	Large-balloon sphincteroplasty-based approach	Successful removal of stones, adverse events, procedure time
Buxbaum (2018) [38]	60 patients	Randomized clinical trial	Patients with an extrahepatic duct stone (common biliary or common hepatic) greater than 1 cm in diameter	United States	Cholangiography-guided laser lithotripsy	Conventional therapeutic techniques, including mechanical basket lithotripsy, papillary dilatation and balloon extraction, or balloon or basket extraction (without lithotripsy) without papillary dilatation	Successful stone removal, adverse events, procedure time
Franzini, (2018) [39]	100 patients	Randomized clinical trial	Patients with difficult biliary stones	Brazil	SOC performed with the first generation of the SpyGlass™ plus EHL system	Large-balloon endoscopic papillary dilation	Successful stone removal, adverse events, procedure time

*DSOC* digital single-operator cholangioscopy, *EHL* electrohydraulic lithotripsy, *ERCP* Endoscopic Retrograde Cholangiopancreatography, *IBS* indeterminate biliary strictures, *SOC* single-operator cholangioscopy

### Diagnosis of biliary strictures of undetermined etiology in adult patients

Should single-operator cholangioscopy (SOC) with stricture pattern characterization and biopsy vs. endoscopic retrograde cholangiopancreatography (ERCP) with brushing and/or biopsy be used for the diagnosis of biliary strictures of undetermined etiology in adult patients?

#### Recommendation 1

For adult patients with primary biliary strictures of undetermined etiology, the panel **suggests** the use of single-operator cholangioscopy (SOC) with the characterization of the stricture pattern and biopsy over endoscopic retrograde cholangiography (ERCP) (*conditional recommendation* for SOC based on *moderate* certainty in the evidence of diagnostic accuracy studies ⊕⊕⊕◯*, and low* certainty in the evidence of effects on clinical outcomes ⊕⊕◯◯).

#### Summary of evidence

We included two relevant primary studies with 143 patients (including 178 specimens) to inform estimates or test accuracy [5, 7]. Furthermore, we identified two systematic reviews that included the two primary studies identified [33, 34]. The EtD framework is shown online at: https://guidelines.gradepro.org/profile/L4UnXKg8Fjk.

#### Benefits, harms, and burden

Moderate certainty evidence showed that SOC with a characterization of the stenosis pattern and biopsies had a pooled sensitivity of 64,9% (95% CI 48.5–78.4%) and a pooled specificity of 100% (95% CI 0–100%) compared to ERCP with brushing and/or biopsy (sensitivity: 51%, 95% CI 14–86%, and specificity 100%, 95% CI 0–100%) [5, 7] (Refer to the online EtD framework: https://guidelines.gradepro.org/profile/L4UnXKg8Fjk). Based on a prevalence of 46%, an estimated 162 specimens per 1000 tested would have falsely normal findings with SOC, and 0 specimens per 1000 tested would have falsely abnormal findings with a SOC. The panel felt that the desirable effect of the intervention is of high impact on subsequent management and for consequences in the control and development of the disease. The panel discussed that postoperative cholangitis is one of the potential harms of SOC; however, the risk is not higher than that of ERCP, particularly in patients with cholelithiasis. The risk was considered similar between the two interventions. Other undesirable effects discussed by the panel included bleeding or perforation, but they suggested that the risk remains like ERCP's. Low certainty of evidence shows no difference in the procedure success rate using SOC with the characterization of the stricture pattern and biopsies compared to ERCP with brushing and/or biopsy based on the RCT finding [7] (RR: 1.17, 95% CI 0.90–1.52). The findings of the retrospective cohort study [5] were consistent with the findings of the randomized trial for procedure success rate (OR: 1.08, 95% CI 0.11–10.78). (Refer to the online EtD framework: https://guidelines.gradepro.org/profile/L4UnXKg8Fjk). The panel considered that comparing the two procedures in clinical practice for the procedure success rate is difficult as their effectiveness may depend on the operator's experience and the clinical characteristics of each patient. The overall certainty in the evidence was moderate for diagnostic accuracy studies owing to risk of bias, and low owing to risk of bias and imprecision for the effects on clinical outcomes.

#### Other EtD Criteria and considerations

We did not identify systematic reviews that assessed patients' values and preferences. Based on their expertise, the panel determined that there was no important uncertainty or variability in how much-affected individuals valued the critical outcomes. No included systematic reviews or primary studies reported either on equity, acceptability, or feasibility. No systematic reviews reported on the cost-effectiveness of the reviewed interventions in Colombia. We found one study [35]. that addressed an economic evaluation, without cost-effectiveness evidence, of the use of SOC compared to conventional ERCP from a Belgium hospital perspective. This study favored the use of SOC over ERCP in the total cost analysis mainly due to lower diagnostic costs and shorter hospital stay follow-up [35]. The panel estimated the cost of SOC in Colombia to range between of 2000 to 8,000 USD depending on the institution and the city where the cost evaluation was conducted. We identified one systematic review that reported evidence regarding the baseline risk [36]. The systematic review included 6 studies that included 283 patients that underwent Digital Single-Operator Cholangioscopy (DSOC), and reported a pooled prevalence, interpreted as the pretest prevalence of malignancy, of 46% (95% CI: 40% to 52%). We did not found evidence of baseline risk for Colombia or Latin-American population.

#### Conclusions and research needs for this recommendation

The guideline panel determined that there is moderate certainty in the test accuracy evidence for the use of SOC. Based on the available evidence, SOC is likely to improve diagnostic performance in patients with biliary strictures of undetermined etiology. The balance of desirable and undesirable consequences favors the use of SOC over ERCP in adult patients with biliary strictures of undetermined etiology. Specifically, the panel considered that most patients would choose SOC due to its diagnostic accuracy. The use of cholangioscopy for diagnosing biliary strictures of undetermined etiology is conditioned by the availability of this technology at healthcare centers. When not available, ERCP may be an alternative diagnostic method. Likewise, the diagnostic accuracy of the intervention may be influenced by the patient's condition. Under certain circumstances, patients will likely undergo more than one cholangioscopy procedure and require special monitoring in terms of survival, complications, type, and duration of intervention. The creation of a national database of centers specialized in cholangioscopy is a priority to allow the monitoring of patients, as well as carrying out economic evaluations of the intervention. The EtD framework is shown online at: https://guidelines.gradepro.org/profile/L4UnXKg8Fjk).

### Management of patients with difficult biliary stones

Should single-operator cholangioscopy (SOC) vs. endoscopic retrograde cholangiopancreatography (ERCP) with large-balloon papillary dilation be used for the management of adult patients with difficult biliary stones?

#### Recommendation 2

For adult patients with difficult biliary stones, the panel **suggests** the use of single-operator cholangioscopy (SOC) over endoscopic retrograde cholangiography (ERCP) with large-balloon papilla dilation (*conditional recommendation* for SOC based on *low* certainty in the evidence of effects on clinical outcomes ⊕  ⊕ ◯◯).

####  Summary of evidence

We identified 3 randomized clinical trials [37–39] that met our inclusion criteria. Additionally, we found 3 systematic reviews addressing this question but considering different populations [2, 40, 41]. The primary studies identified by our search were included in these systematic reviews. The EtD framework is shown online at: https://guidelines.gradepro.org/profile/piL0zuqxntA

#### Benefits, harms, and burden

Among critical outcomes, moderate certainty evidence showed that SOC compared to ERCP with large-balloon papillary dilation may result in little to no differences in number of successful therapeutic maneuvers for biliary stone removal (RR 1.25, 95% CI: 0.95—1.63) [37, 39], or procedure time (SMD 0.46, -0.21 – 1.13) [37, 39]. Low certainty evidence showed that associated risk for adverse effects may not differ between SOC ERCP with large-balloon papillary dilation (RR 0.88, 95% CI: 0.11—7.13) [37, 39] (Refer to the online EtD framework: https://guidelines.gradepro.org/profile/piL0zuqxntA). The overall certainty in the evidence for effects was low owing to risk of bias and imprecision on critical outcomes.

#### Other EtD criteria and considerations

We did not find systematic reviews that reported evidence on values and preferences. Based on their clinical expertise, the panel considered there was no important uncertainty or variability on the critical outcomes prioritized by the panel. No included systematic reviews or primary studies reported either on equity, acceptability, or feasibility. We did not identify systematic reviews or primary studies that reported resource use or cost-effectiveness of the interventions in the Colombian context. We found two primary studies [35, 37] that considered the use of resources and the cost of SOC compared to ERCP with large-balloon papillary dilation in the management of difficult biliary stones in a Belgium hospital [35] and an interventional endoscopy in the United States [37], respectively. None of them reported cost-effectiveness data but reported cost analysis of each procedure. The first study favors SOC over ERCP in the total cost analysis due to shorter hospital stay follow-up and reduced number of reinterventions [35]. For the US study, authors reported a higher cost associated with SOC compared to ERCP; however, this difference ended up being unimportant compared with the total costs of the procedures [37]. In the Colombian context, and based on their experience, the panel considered that the cost-effectiveness may favor SOC over ERCP. They reflected that even if SOC as a single procedure may be more expensive than ERCP, a patient may receive several ERCPs before receiving a SOC.

#### Conclusions and research needs for this recommendation

The guideline panel determined that although there is low certainty in the evidence to establish a net balance of health benefit versus harm from the use of SOC, based on the body of evidence available, SOC is likely to improve the number of therapeutic maneuvers for complete difficult biliary stone removal and decrease the incidence of adverse events. The balance of desirable and undesirable consequences favors the use of SOC versus ERCP with large-balloon papillary dilatation in patients with difficult biliary stones. The panel considered that most patients would choose SOC due to the therapeutic success of this technique and the number of successful maneuvers. The panel identified the need for high-quality economic evaluation studies in areas such as cost-effectiveness and use of resources required, generation of hospital indicators in the Colombian health system context related to the implementation of SOC, as well as quality management of cholangioscopy procedures. The EtD framework is shown online at: https://guidelines.gradepro.org/profile/piL0zuqxntA.

## Discussion

This guideline scope is distinctive because it covers all widespread SOC and ERCP-related issues. Every recommendation includes a formal EtD framework based on high-quality systematic reviews, which improves the judgments' transparency. The National Institute for Health and Care Excellence (NICE) [6] in 2015 evaluated the SpyGlass system for the diagnostic and therapeutic management of biliary system diseases, suggesting that SpyGlass should be used when standard techniques are unsuccessful or inappropriate. Unlike standard ERCP, SpyGlass is a single-operator system designed to visualize and facilitate access to the bile ducts during diagnostic and therapeutic procedures. The SpyGlass system is intended for use in endoscopic units with the equipment and expert personnel to perform ERCP [6].

The International Hepato-Pancreato-Biliary Association [8] agreed on the clinical role of cholangioscopy in the diagnosis of indeterminate biliary stenosis. International experts reviewed the evidence and made the statements using a consensus method, defining that, when available, cholangioscopy evaluation and guided biopsy during the first round of ERCP can reduce the need for multiple procedures. Additionally, the experts considered that direct peroral cholangioscopy provides the largest accessory channel, better image definition, and is technically more demanding than conventional methods. The panel concluded that cholangioscopy is complementary to abdominal imaging and ERCP tissue acquisition in evaluating and diagnosing indeterminate biliary strictures [8].

The European Society of Gastrointestinal Endoscopy (ESGE) guideline [15], endorses the use of SOC-assisted intraluminal lithotripsy as an effective and safe treatment for challenging bile duct stones. Furthermore, the ESGE suggests that the choice of cholangioscopy and lithotripsy should be based on local availability and expertise. This underscores the importance of locally focused guidelines, which tailor recommendations to the distinctive circumstances of each setting for improved outcomes.

Our CPG differs from the NICE [5], the International Hepato-Pancreato-Biliary Association reports [8], and the ESGE guideline [15] in discussing the strength of the evidence and in the inclusion of patients’ values and preferences for recommendations when an optimal treatment option is lacking. This empowers patients and their providers to make decisions based on personal health history, preferences, and values with the best evidence available nowadays.

## Limitations of this CPG

The limitations of these guidelines are inherent to the moderate or low certainty in the evidence identified for the prioritized questions. The panel acknowledges that there is insufficient evidence related to SOC with stricture pattern characterization and biopsies versus ERCP with brushing and/or biopsy for the diagnosis of biliary stricture of undetermined etiology, raising questions about the applicability of the evidence obtained in terms of diagnostic performance the Colombian context.

We used evidence from a metanalysis [36] to determine the baseline risks of patients with biliary stricture of undetermined etiology. However, it includes six studies with a low total number of patients (283 patients), which could reduce the applicability of the information to the Colombian context.

Considering the evidence of the use of resources and cost-effectiveness, only one article was obtained [35]. The information reported in that document came from European population. Therefore, the extrapolation of these data to the Colombian context is challenging. Nevertheless, although the implementation of SOC requires an initial considerable financing in equipment, technology, and user training, the clinical benefits and the reduction of additional reinterventions outweigh the initial investment [34]. Due to SOC's improved diagnostic accuracy, decreased reintervention procedure needs, and adverse outcomes, as well as faster recovery and shorter hospital stays, the use of SOC generally results in a favorable reduction of costs associated with both the diagnosis of indeterminate biliary strictures and the management of difficult biliary stones over the long term [35].

In addition, the panel acknowledges that, for aspects such as values and preferences, and other contextual factors, it was not possible to obtain articles aligned with the objective of this guideline, which led the experts to make decisions based on their experience. This enriches the content related to the recommendations but could limit its value when making decisions from a population perspective.

As monitoring strategy this guideline will be updated by the ACED in 2 years or before if there is new of evidence or updated recommendations from other guidelines that modify the strength and direction of these recommendations.

## Conclusion

The scope of this CPG includes questions relevant to the diagnosis of indeterminate biliary stricture and the treatment of difficult biliary stones using SOC. We conducted high-quality systematic reviews and provided a formal EtD framework for each recommendation, increasing the transparency of the assessments made. The findings of this guideline target national use, with local clinical practice availability determining the extent to which they can be applied. While global evidence supports regional application, it is crucial to assess the implications within the diverse health systems of the region.

### Supplementary Information

Below is the link to the electronic supplementary material.

Supplementary file1 (DOCX 16 kb)

Supplementary file2 (DOCX 15 kb)

Supplementary file3 (DOCX 29 kb)

Supplementary file4 (DOCX 90 kb)

## Abbreviations

ACED: Colombian Association of Digestive Endoscopy (ACED from the Spanish initials for Asociación Colombiana de Endoscopia Digestiva)
CPG: Clinical practice guide
DSOC: Digital single-operator cholangioscopy
EHL: Electrohydraulic lithotripsy
ERCP: Endoscopic retrograde cholangiopancreatography
EtD: Evidence to decision framework
GIN: Guidelines International Network
GRADE: Grading of Recommendations Assessment, Development, and Evaluation
IBS: Indeterminate biliary strictures
IOM: Institute of Medicine
MRI: Magnetic resonance imaging
NICE: National Institute for Health and Care Excellence
OR: Odds ratios
QUADAS: A revised tool for the quality assessment of diagnostic accuracy studies
RR: Relative risk
SD: Standard deviation
SOC: Single-operator cholangioscopy
WHO: World Health Organization
