# Supplementary material for: Clinical practice guideline on the use of single-operator cholangioscopy in the diagnosis of indeterminate biliary stricture and the treatment of difficult biliary stones
Source: Surg Endosc. 2023 Dec 26;38(2):499–510. doi: 10.1007/s00464-023-10569-x (PMC10830582; doi:10.1007/s00464-023-10569-x)
Supplement: Supplementary file 1 — Supplementary file1 (DOCX 16 kb) [file 464_2023_10569_MOESM1_ESM.docx]

**Supplementary material 1. Description of the group of participants in the panel of experts and the reviewer group in charge of developing the guideline**

| **Name** |  | **Participation in diagnostic question** | **Participation in treatment question** | **Role in CPG** |
| --- | --- | --- | --- | --- |
| ***Panel experts*** | | | | |
| Lazaro Antonio Arango Molano | Surgeon/ Gastroenterology, endoscopy and ERCP | Yes | Yes | Panel expert |
| Camilo Blanco-Avellaneda | Surgeon/ Gastroenterology, Endoscopy | Yes | Yes | Panel expert |
| Jhon Jaime Carvajal Gutiérrez | Internist /Gastroenterology, endoscopy and ERCP | Yes | Yes | Panel expert |
| Rodrigo Castaño-Llano | Surgeon/ Gastroenterology, endoscopy and ERCP | Yes | Yes | Panel expert |
| Martin Alonso Gómez Zuleta | Internist /Gastroenterology, endoscopy and ERCP | Yes | Yes | Panel expert |
| Carlos A González S | Surgeon/ Gastroenterology, endoscopy and ERCP | Yes | Yes | Panel expert |
| Arecio Peñaloza- Ramírez | Internist /Gastroenterology, endoscopy and ERCP | Yes | Yes | Panel expert |
| Raúl Pinilla Morales | Surgeon/ Gastroenterology, endoscopy and ERCP | Yes | Yes | Panel expert |
| Renzo Pinto Carta | Internist /Gastroenterology, endoscopy and ERCP | Yes | Yes | Panel expert |
| Héctor Adolfo Polanía Liscano | Surgeon/ Gastroenterology, endoscopy and ERCP | Yes | Yes | Panel expert |
| Adriana Margarita Rey Rubiano | Internist /Gastroenterologist, Endoscopy | Yes | Yes | Panel expert leader |
| Reinaldo Andrés Rincón Sánchez | Internist /Gastroenterology, endoscopy and ERCP | Yes | Yes | Panel expert |
| Mauricio Sepúlveda Copete | Internist /Gastroenterology, endoscopy and ERCP | No | Yes | Panel expert |
| Rómulo Vargas-Rubio | Internist /Gastroenterology, endoscopy and ERCP | Yes | Yes | Panel expert |
| ***Reviewer team*** | | | | |
| Camilo Andrés Avendaño Capriles | Medical doctor | Yes | Yes | Researcher |
| Andrés Mauricio García Sierra | Medical doctor, master’s degree in public health and doctorate candidate | Yes | Yes | Researcher |
| Laura Yuriko González Teshima | Medical doctor, epidemiology specialist | No | Yes | Researcher |
| Juan José Yepes Nuñez | Medical doctor, master's degree, and a doctorate in clinical epidemiology | Yes | Yes | Methodological leader |
