# Supplementary material for: Clinical practice guideline on the use of single-operator cholangioscopy in the diagnosis of indeterminate biliary stricture and the treatment of difficult biliary stones
Source: Surg Endosc. 2023 Dec 26;38(2):499–510. doi: 10.1007/s00464-023-10569-x (PMC10830582; doi:10.1007/s00464-023-10569-x)
Supplement: Supplementary file 2 — Supplementary file2 (DOCX 15 kb) [file 464_2023_10569_MOESM2_ESM.docx]

**Supplementary material 2. Declaration of conflict of interest of participants of the guideline**

| ***Name*** | ***Form filing date*** | ***Type of declaration*** | ***Declaration*** |
| --- | --- | --- | --- |
| Lázaro Antonio Arango Molano | 15/12/2021 | Employment and consulting  In the last 4 years, have you received any compensation from any commercial entity or other organization with an interest related to the subject of the meeting or work? | Consulting, including serving as a technical or other adviser to companies nonrelated to the topic of this guideline. |
| Camilo Blanco-Avellaneda | 07/01//2022 | None | No conflicts |
| Jhon Jaime Carvajal Gutiérrez | 07/01/2022 | None | No conflicts |
| Rodrigo Castaño-Llano | 21/12/2021 | None | No conflicts |
| Martin Alonso Gómez Zuleta | 15/12/2021 | Employment and consulting  In the last 4 years, have you received any compensation from any commercial entity or other organization with an interest related to the subject of the meeting or work? | Consulting, including serving as a technical or other adviser |
| Carlos A González S | 21/02/2022 | Research support  In the last 4 years, have you or your research unit received support from a commercial entity or other organization with an interest related to the topic of the meeting or the work? | Research support, including donations, collaborations, sponsorships, and other funding sources.  Non-monetary support, valued generally at more than US$1,000 (include equipment, facilities, research assistants, paid travel to meetings, etc.) from companies nonrelated to the topic of this guideline. |
| Arecio Peñaloza- Ramírez | 03/01/2022 | Have you received any payment (other than travel expenses) or fees for speaking publicly on the subject of this meeting or WHO's work? | Yes, however all public speeches were nonrelated to the topic of this guideline specifically. |
| Raúl Pinilla Morales | 29/12/2021 | Public statements and positions (during the last 3 years)  Have you held any position or other position, paid or not, in which you represented interests or defended a position related to the subject of the meeting or work? | Yes, however all public statements are positions were nonrelated to the topic of this guideline specifically. |
| Renzo Pinto Carta | 20/12/2021 | None | No conflicts |
| Héctor Adolfo Polanía Liscano | 15/12/2021 | None | No conflicts |
| Adriana Margarita Rey Rubiano | 21/12/2021 | None | No conflicts |
| Reinaldo Andrés Rincón Sánchez | 17/12/2021 | None | No conflicts |
| Mauricio Sepúlveda Copete | 17/12/2021 | None | No conflicts |
| Rómulo Vargas-Rubio | 15/12/2021 | None | No conflicts |
| Camilo Andrés Avendaño Capriles | 15/12/2021 | None | No conflicts |
| Andrés Mauricio García Sierra | 15/12/2021 | None | No conflicts |
| Laura Yuriko González Teshima | 15/12/2021 | None | No conflicts |
| Juan José Yepes Nuñez | 15/12/2021 | None | No conflicts |
