# Supplementary material for: Clinical practice guideline on the use of single-operator cholangioscopy in the diagnosis of indeterminate biliary stricture and the treatment of difficult biliary stones
Source: Surg Endosc. 2023 Dec 26;38(2):499–510. doi: 10.1007/s00464-023-10569-x (PMC10830582; doi:10.1007/s00464-023-10569-x)
Supplement: Supplementary file 3 — Supplementary file3 (DOCX 29 kb) [file 464_2023_10569_MOESM3_ESM.docx]

**Supplementary material 3. Search strategies**

| ***Treatment and diagnosis*** |
| --- |
| **Interface: Ovid**  **Database: Medline**  **Type of studies: No restriction** |
| 1 ERCP.mp.  2 exp endoscopic retrograde cholangiopancreatography/  3 (endoscop$ adj3 retrograd$ adj3 cholangiopancreatograph$).tw.  4 exp Sphincterotomy, Endoscopic/  5 (endoscopic adj3 sphincterotom$).mp.  6 papillotomy.mp.  7 or/1-6  8 CT.ti,ab.  9 tomodensitometry.ti,ab.  10 MRI.ti,ab.  11 NMRI.ti,ab.  12 zeugmatogra*.ti,ab.  13 computed.ti,ab.  14 computerised.ti,ab.  15 computerized.ti,ab.  16 magneti*.ti,ab.  17 MR.ti,ab.  18 NMR.ti,ab.  19 proton.ti,ab.  20 tomogra*.ti,ab.  21 scan.ti,ab.  22 scans.ti,ab.  23 imaging.ti,ab.  24 cholangiogra*.ti,ab.  25 exp Tomography, X-Ray Computed/  26 exp Magnetic Resonance Imaging/  27 cholangiogra*.ti,ab.  28 cholangio?pancreatogra*.ti,ab.  29 cholangiosco*.ti,ab.  30 choledochosco*.ti,ab.  31 ERCP.ti,ab.  32 MRCP.ti,ab.  33 exp Cholangiography/  34 exp Cholangiopancreatography, Magnetic Resonance/  35 8 or 9 or 10 or 11 or 12 or 13 or 14 or 15 or 16 or 17 or 18 or 19 or 20 or 21 or 22 or 23 or 24 or 25 or 26 or 27 or 28 or 29 or 30 or 31 or 32 or 33 or 34  36 Endoscopy, Digestive System/  37 cholangioscopy.mp.  38 Cholangiopancreatography, Endoscopic Retrograde/  39 cholangiopancreatoscopy.mp.  40 cholecystoscopy.mp.  41 ercp.mp.  42 36 or 37 or 38 or 39 or 40 or 41  43 single operator.mp.  44 direct visuali?ation.mp. [mp=title, abstract, original title, name of substance word, subject heading word, floating sub-heading word, keyword heading word, organism supplementary concept word, protocol supplementary concept word, rare disease supplementary concept word, unique identifier, synonyms]  45 43 or 44  46 42 and 45  47 spyglass.mp. [mp=title, abstract, original title, name of substance word, subject heading word, floating sub-heading word, keyword heading word, organism supplementary concept word, protocol supplementary concept word, rare disease supplementary concept word, unique identifier, synonyms]  48 46 or 47  49 remove duplicates from 48  50 7 or 35  51 49 and 50  52 limit 51 to (english language and humans)53  53 limit 52to yr="2014 -2022" |

| ***Treatment and diagnosis*** |
| --- |
| **Interface: Embase**  **Database: Embase**  **Type of studies: No restriction** |
| 1 '(('ercp' OR 'endoscopic retrograde cholangiopancreatography/' OR 'endoscop$ retrograd$ cholangiopancreatograph$' OR 'sphincterotomy, endoscopic/' OR 'endoscopic sphincterotom$' OR 'papillotomy') AND ('randomized controlled trial' OR 'controlled clinical trial' OR 'random$' OR trial OR groups) OR ((proton:ti,ab OR nmr:ti,ab OR mr:ti,ab OR magneti*:ti,ab OR computarized:ti,ab OR computarised:ti,ab OR computed:ti,ab) AND (cholangiogra*:ti,ab OR imaging:ti,ab OR scans:ti,ab OR scan:ti,ab OR tomogra*:ti,ab)) OR zeugmatogra*:ti,ab OR nmri:ti,ab OR mri:ti,ab OR tomodensitometry:ti,ab OR ct:ti,ab OR 'cholangiopancreatography, magnetic resonance' OR choledochosco*:ti,ab OR 'cholangiography' OR mrcp:ti,ab OR ercp:ti,ab OR cholledochosco*:ti,ab OR cholangiosco*:ti,ab OR cholangio?pancreatogra*:ti,ab OR cholangiogra*:ti,ab OR 'magnetic resonance imaging' OR 'tomography, x-ray computed') AND (('endoscopy, digestive system/' OR 'cholangioscopy' OR 'cholangiopancreatography, endoscopic retrograde/' OR 'cholangiopancreatoscopy' OR 'cholecystoscopy' OR 'ercp') AND ('single operator' OR 'direct visuali?ation') OR 'spyglass')  2 1 AND [english]/lim AND [humans]/lim AND [embase]/lim AND [2014-2022]/py |

| ***Baseline risks*** |
| --- |
| **Interface: Ovid**  **Database: Medline**  **Type of studies: No restriction** |
| 1 ERCP.mp.  2 exp endoscopic retrograde cholangiopancreatography/  3 (endoscop$ adj3 retrograd$ adj3 cholangiopancreatograph$).tw.  4 exp Sphincterotomy, Endoscopic/  5 (endoscopic adj3 sphincterotom$).mp.  6 papillotomy.mp.  7 or/1-6  8 CT.ti,ab.  9 tomodensitometry.ti,ab.  10 MRI.ti,ab.  11 NMRI.ti,ab.  12 zeugmatogra*.ti,ab.  13 computed.ti,ab.  14 computerised.ti,ab.  15 computerized.ti,ab.  16 magneti*.ti,ab.  17 MR.ti,ab.  18 NMR.ti,ab.  19 proton.ti,ab.  20 tomogra*.ti,ab.  21 scan.ti,ab.  22 scans.ti,ab.  23 imaging.ti,ab.  24 cholangiogra*.ti,ab.  25 exp Tomography, X-Ray Computed/  26 exp Magnetic Resonance Imaging/  27 cholangiogra*.ti,ab.  28 cholangio?pancreatogra*.ti,ab.  29 cholangiosco*.ti,ab.  30 choledochosco*.ti,ab.  31 ERCP.ti,ab.  32 MRCP.ti,ab.  33 exp Cholangiography/  34 exp Cholangiopancreatography, Magnetic Resonance/  35 8 or 9 or 10 or 11 or 12 or 13 or 14 or 15 or 16 or 17 or 18 or 19 or 20 or 21 or 22 or 23 or 24 or 25 or 26 or 27 or 28 or 29 or 30 or 31 or 32 or 33 or 34  36 Endoscopy, Digestive System/  37 cholangioscopy.mp.  38 Cholangiopancreatography, Endoscopic Retrograde/  39 cholangiopancreatoscopy.mp.  40 cholecystoscopy.mp.  41 ercp.mp.  42 36 or 37 or 38 or 39 or 40 or 41  43 single operator.mp.  44 direct visuali?ation.mp. [mp=title, abstract, original title, name of substance word, subject heading word, floating sub-heading word, keyword heading word, organism supplementary concept word, protocol supplementary concept word, rare disease supplementary concept word, unique identifier, synonyms]  45 43 or 44  46 42 and 45  47 spyglass.mp. [mp=title, abstract, original title, name of substance word, subject heading word, floating sub-heading word, keyword heading word, organism supplementary concept word, protocol supplementary concept word, rare disease supplementary concept word, unique identifier, synonyms]  48 46 or 47  49 remove duplicates from 48  50 7 or 35  51 49 and 50  52 (exp *Epidemiological Monitoring/ or exp *Incidence/ or ep,eh,mp.fs. or exp *Population Surveillance/ or exp *Prevalence/ or denominator.ti,ab,cl,oa,kw,kf. or epidemiolog*.ti,bt. or frequency.ti,bt. or incident.ti,bt. or incidence.ti,ab,cl,oa,kw,kf. or population-based.ti,ab,cl,oa,kw,kf. or prevalent.ti,bt. or prevalence.ti,ab,cl,oa,kw,kf. or proportion.ti,bt. or rate.ti,bt. or surveillance.ti,ab,cl,oa,kw,kf.)  53 51 AND 52  54 limit 53 to yr="2006 - 2022"  55 limit 54 to (english language and humans) |

| ***Baseline risks*** |
| --- |
| **Interface: Embase**  **Database: Embase**  **Type of studies: No restriction** |
| 1 '(('ercp' OR 'endoscopic retrograde cholangiopancreatography/' OR 'endoscop$ retrograd$ cholangiopancreatograph$' OR 'sphincterotomy, endoscopic/' OR 'endoscopic sphincterotom$' OR 'papillotomy') AND ('randomized controlled trial' OR 'controlled clinical trial' OR 'random$' OR trial OR groups) OR ((proton:ti,ab OR nmr:ti,ab OR mr:ti,ab OR magneti*:ti,ab OR computarized:ti,ab OR computarised:ti,ab OR computed:ti,ab) AND (cholangiogra*:ti,ab OR imaging:ti,ab OR scans:ti,ab OR scan:ti,ab OR tomogra*:ti,ab)) OR zeugmatogra*:ti,ab OR nmri:ti,ab OR mri:ti,ab OR tomodensitometry:ti,ab OR ct:ti,ab OR 'cholangiopancreatography, magnetic resonance' OR choledochosco*:ti,ab OR 'cholangiography' OR mrcp:ti,ab OR ercp:ti,ab OR cholledochosco*:ti,ab OR cholangiosco*:ti,ab OR cholangio?pancreatogra*:ti,ab OR cholangiogra*:ti,ab OR 'magnetic resonance imaging' OR 'tomography, x-ray computed') AND (('endoscopy, digestive system/' OR 'cholangioscopy' OR 'cholangiopancreatography, endoscopic retrograde/' OR 'cholangiopancreatoscopy' OR 'cholecystoscopy' OR 'ercp') AND ('single operator' OR 'direct visuali?ation') OR 'spyglass')  2 ('epidemiological monitoring'/exp/mj OR 'incidence'/exp/mj OR 'population surveillance'/exp/mj OR 'prevalence'/exp OR denominator:ti,ab OR epidemiolog*:ti OR frequency:ti OR incident:ti OR incidence:ti,ab OR 'population-based':ti,ab OR prevalent:ti OR prevalence:ti,ab OR proportion:ti OR rate:ti OR surveillance:ti,ab)  3 1 AND 2  4 3 AND [english]/lim AND [humans]/lim AND [embase]/lim AND [2006-2022]/py |

| ***Cost-effectiveness*** |
| --- |
| **Interface: Ovid**  **Database: Medline**  **Type of studies: No restriction** |
| 1 ERCP.mp.  2 exp endoscopic retrograde cholangiopancreatography/  3 (endoscop$ adj3 retrograd$ adj3 cholangiopancreatograph$).tw.  4 exp Sphincterotomy, Endoscopic/  5 (endoscopic adj3 sphincterotom$).mp.  6 papillotomy.mp.  7 or/1-6  8 CT.ti,ab.  9 tomodensitometry.ti,ab.  10 MRI.ti,ab.  11 NMRI.ti,ab.  12 zeugmatogra*.ti,ab.  13 computed.ti,ab.  14 computerised.ti,ab.  15 computerized.ti,ab.  16 magneti*.ti,ab.  17 MR.ti,ab.  18 NMR.ti,ab.  19 proton.ti,ab.  20 tomogra*.ti,ab.  21 scan.ti,ab.  22 scans.ti,ab.  23 imaging.ti,ab.  24 cholangiogra*.ti,ab.  25 exp Tomography, X-Ray Computed/  26 exp Magnetic Resonance Imaging/  27 cholangiogra*.ti,ab.  28 cholangio?pancreatogra*.ti,ab.  29 cholangiosco*.ti,ab.  30 choledochosco*.ti,ab.  31 ERCP.ti,ab.  32 MRCP.ti,ab.  33 exp Cholangiography/  34 exp Cholangiopancreatography, Magnetic Resonance/  35 8 or 9 or 10 or 11 or 12 or 13 or 14 or 15 or 16 or 17 or 18 or 19 or 20 or 21 or 22 or 23 or 24 or 25 or 26 or 27 or 28 or 29 or 30 or 31 or 32 or 33 or 34  36 Endoscopy, Digestive System/  37 cholangioscopy.mp.  38 Cholangiopancreatography, Endoscopic Retrograde/  39 cholangiopancreatoscopy.mp.  40 cholecystoscopy.mp.  41 ercp.mp.  42 36 or 37 or 38 or 39 or 40 or 41  43 single operator.mp.  44 direct visuali?ation.mp. [mp=title, abstract, original title, name of substance word, subject heading word, floating sub-heading word, keyword heading word, organism supplementary concept word, protocol supplementary concept word, rare disease supplementary concept word, unique identifier, synonyms]  45 43 or 44  46 42 and 45  47 spyglass.mp. [mp=title, abstract, original title, name of substance word, subject heading word, floating sub-heading word, keyword heading word, organism supplementary concept word, protocol supplementary concept word, rare disease supplementary concept word, unique identifier, synonyms]  48 46 or 47  49 remove duplicates from 48  50 7 or 35  51 49 and 50  52 "costs and cost analysis"[MeSH Terms]  53 "costs"[Title/Abstract]  54 "cost effective*"[Title/Abstract]  55 "cost*"[Title/Abstract]  56 "costs and cost analysis"[MeSH Terms:noexp]  57 "cost benefit analys*"[Title/Abstract]  58 "cost benefit analysis"[MeSH Terms]  59 health care costs"[MeSH Terms:noexp]  60 or/55-59  61 52 or 53 or 54 or 60  62 51 AND 61  63 limit 62 to yr="2006 - 2022"  55 limit 63 to (english language and humans) |

| ***Cost-effectiveness*** |
| --- |
| **Interface: Embase**  **Database: Embase**  **Type of studies: No restriction** |
| 1 '(('ercp' OR 'endoscopic retrograde cholangiopancreatography/' OR 'endoscop$ retrograd$ cholangiopancreatograph$' OR 'sphincterotomy, endoscopic/' OR 'endoscopic sphincterotom$' OR 'papillotomy') AND ('randomized controlled trial' OR 'controlled clinical trial' OR 'random$' OR trial OR groups) OR ((proton:ti,ab OR nmr:ti,ab OR mr:ti,ab OR magneti*:ti,ab OR computarized:ti,ab OR computarised:ti,ab OR computed:ti,ab) AND (cholangiogra*:ti,ab OR imaging:ti,ab OR scans:ti,ab OR scan:ti,ab OR tomogra*:ti,ab)) OR zeugmatogra*:ti,ab OR nmri:ti,ab OR mri:ti,ab OR tomodensitometry:ti,ab OR ct:ti,ab OR 'cholangiopancreatography, magnetic resonance' OR choledochosco*:ti,ab OR 'cholangiography' OR mrcp:ti,ab OR ercp:ti,ab OR cholledochosco*:ti,ab OR cholangiosco*:ti,ab OR cholangio?pancreatogra*:ti,ab OR cholangiogra*:ti,ab OR 'magnetic resonance imaging' OR 'tomography, x-ray computed') AND (('endoscopy, digestive system/' OR 'cholangioscopy' OR 'cholangiopancreatography, endoscopic retrograde/' OR 'cholangiopancreatoscopy' OR 'cholecystoscopy' OR 'ercp') AND ('single operator' OR 'direct visuali?ation') OR 'spyglass')  2 ('cost effective*' OR 'health care costs' OR 'cost benefit analysis' OR 'cost benefit analys*' OR 'cost*' OR 'cost effective' OR 'costs' OR 'costs and cost analysis')  3 1 AND 2  4 3 AND [english]/lim AND [humans]/lim AND [embase]/lim AND [2006-2022]/py |

| ***Values and preferences*** |
| --- |
| **Interface: Ovid**  **Database: Medline**  **Type of studies: No restriction** |
| 1 ERCP.mp.  2 exp endoscopic retrograde cholangiopancreatography/  3 (endoscop$ adj3 retrograd$ adj3 cholangiopancreatograph$).tw.  4 exp Sphincterotomy, Endoscopic/  5 (endoscopic adj3 sphincterotom$).mp.  6 papillotomy.mp.  7 or/1-6  8 CT.ti,ab.  9 tomodensitometry.ti,ab.  10 MRI.ti,ab.  11 NMRI.ti,ab.  12 zeugmatogra*.ti,ab.  13 computed.ti,ab.  14 computerised.ti,ab.  15 computerized.ti,ab.  16 magneti*.ti,ab.  17 MR.ti,ab.  18 NMR.ti,ab.  19 proton.ti,ab.  20 tomogra*.ti,ab.  21 scan.ti,ab.  22 scans.ti,ab.  23 imaging.ti,ab.  24 cholangiogra*.ti,ab.  25 exp Tomography, X-Ray Computed/  26 exp Magnetic Resonance Imaging/  27 cholangiogra*.ti,ab.  28 cholangio?pancreatogra*.ti,ab.  29 cholangiosco*.ti,ab.  30 choledochosco*.ti,ab.  31 ERCP.ti,ab.  32 MRCP.ti,ab.  33 exp Cholangiography/  34 exp Cholangiopancreatography, Magnetic Resonance/  35 8 or 9 or 10 or 11 or 12 or 13 or 14 or 15 or 16 or 17 or 18 or 19 or 20 or 21 or 22 or 23 or 24 or 25 or 26 or 27 or 28 or 29 or 30 or 31 or 32 or 33 or 34  36 Endoscopy, Digestive System/  37 cholangioscopy.mp.  38 Cholangiopancreatography, Endoscopic Retrograde/  39 cholangiopancreatoscopy.mp.  40 cholecystoscopy.mp.  41 ercp.mp.  42 36 or 37 or 38 or 39 or 40 or 41  43 single operator.mp.  44 direct visuali?ation.mp. [mp=title, abstract, original title, name of substance word, subject heading word, floating sub-heading word, keyword heading word, organism supplementary concept word, protocol supplementary concept word, rare disease supplementary concept word, unique identifier, synonyms]  45 43 or 44  46 42 and 45  47 spyglass.mp. [mp=title, abstract, original title, name of substance word, subject heading word, floating sub-heading word, keyword heading word, organism supplementary concept word, protocol supplementary concept word, rare disease supplementary concept word, unique identifier, synonyms]  48 46 or 47  49 remove duplicates from 48  50 7 or 35  51 49 and 50  52 (exp *Decision Making/ or "avoidance behavi*".ti,ab,cl,oa,kw,kf. or "avoidance learning".ti,ab,cl,oa,kw,kf. or decision*.ti,ab,cl,oa,kw,kf. or "decision aid*".ti,ab,cl,oa,kw,kf. or "decision analy*".ti,ab,cl,oa,kw,kf. or "decision board*".ti,ab,cl,oa,kw,kf. or "decision mak*".ti,ab,cl,oa,kw,kf. or "decisions mak*".ti,ab,cl,oa,kw,kf. or decision-support.ti,ab,cl,oa,kw,kf. or "decision tool*".ti,ab,cl,oa,kw,kf. or "discrete choice".ti,ab,cl,oa,kw,kf. or discrete-choice*.ti,ab,cl,oa,kw,kf. or (decision*.ti,bt. and making.ti,bt.))  53 (exp *Attitude to Health/ or exp *Patient Participation/ or exp *Patient Satisfaction/ or choice*.ti,bt. or valuat*.ti,bt. or value*.ti,bt. or acceptab*.ti,ab,cl,oa,kw,kf. or attitude*.ti,ab,cl,oa,kw,kf. or expectation*.ti,ab,cl,oa,kw,kf. or "health perception*".ti,ab,cl,oa,kw,kf. or "health state values".ti,ab,cl,oa,kw,kf. or "health values".ti,ab,cl,oa,kw,kf. or knowledge.ti,ab,cl,oa,kw,kf. or "patient choice*".ti,ab,cl,oa,kw,kf. or "patient participation".ti,ab,cl,oa,kw,kf. or "patient perce*".ti,ab,cl,oa,kw,kf. or "patient perspective*".ti,ab,cl,oa,kw,kf. or "patient valuat*".ti,ab,cl,oa,kw,kf. or "patient value*".ti,ab,cl,oa,kw,kf. or "patient view*".ti,ab,cl,oa,kw,kf. or "patients choice*".ti,ab,cl,oa,kw,kf. or "patients participation".ti,ab,cl,oa,kw,kf. or "patients perce*".ti,ab,cl,oa,kw,kf. or "patients perspective*".ti,ab,cl,oa,kw,kf. or "patients valuat*".ti,ab,cl,oa,kw,kf. or "patients value*".ti,ab,cl,oa,kw,kf. or "patients view*".ti,ab,cl,oa,kw,kf. or "patients' choice*".ti,ab,cl,oa,kw,kf. or "patients' participation".ti,ab,cl,oa,kw,kf. or "patients' perce*".ti,ab,cl,oa,kw,kf. or "patients' perspective*".ti,ab,cl,oa,kw,kf. or "patients' valuat*".ti,ab,cl,oa,kw,kf. or "patients' value*".ti,ab,cl,oa,kw,kf. or "patients' view*".ti,ab,cl,oa,kw,kf. or "patient's choice*".ti,ab,cl,oa,kw,kf. or "patient's participation".ti,ab,cl,oa,kw,kf. or "patient's perce*".ti,ab,cl,oa,kw,kf. or "patient's perspective*".ti,ab,cl,oa,kw,kf. or "patient's valuat*".ti,ab,cl,oa,kw,kf. or "patient's value*".ti,ab,cl,oa,kw,kf. or "patient's view*".ti,ab,cl,oa,kw,kf. or preference*.ti,ab,cl,oa,kw,kf. or "user choice*".ti,ab,cl,oa,kw,kf. or "user participation".ti,ab,cl,oa,kw,kf. or "user perce*".ti,ab,cl,oa,kw,kf. or "user perspective*".ti,ab,cl,oa,kw,kf. or "user valuat*".ti,ab,cl,oa,kw,kf. or "user value*".ti,ab,cl,oa,kw,kf. or "user view*".ti,ab,cl,oa,kw,kf. or "users choice*".ti,ab,cl,oa,kw,kf. or "users participation".ti,ab,cl,oa,kw,kf. or "users perce*".ti,ab,cl,oa,kw,kf. or "users perspective*".ti,ab,cl,oa,kw,kf. or "users valuat*".ti,ab,cl,oa,kw,kf. or "users value*".ti,ab,cl,oa,kw,kf. or "users view*".ti,ab,cl,oa,kw,kf. or "users' choice*".ti,ab,cl,oa,kw,kf. or "users' participation".ti,ab,cl,oa,kw,kf. or "users' perce*".ti,ab,cl,oa,kw,kf. or "users' perspective*".ti,ab,cl,oa,kw,kf. or "users' valuat*".ti,ab,cl,oa,kw,kf. or "users' value*".ti,ab,cl,oa,kw,kf. or "users' view*".ti,ab,cl,oa,kw,kf. or "user's choice*".ti,ab,cl,oa,kw,kf. or "user's participation".ti,ab,cl,oa,kw,kf. or "user's perce*".ti,ab,cl,oa,kw,kf. or "user's perspective*".ti,ab,cl,oa,kw,kf. or "user's valuat*".ti,ab,cl,oa,kw,kf. or "user's value*".ti,ab,cl,oa,kw,kf. or "user's view*".ti,ab,cl,oa,kw,kf.)  54 (exp Choice Behavior/ or exp Decision Making/ or exp Decision Support Systems, Clinical/ or exp Decision Support Techniques/ or (health.ti,bt. and utilit*.ti,bt.) or "best worst".ti,ab,cl,oa,kw,kf. or "best-worst scaling".ti,ab,cl,oa,kw,kf. or "feeling thermometer*".ti,ab,cl,oa,kw,kf. or gamble*.ti,ab,cl,oa,kw,kf. or "health state".ti,ab,cl,oa,kw,kf. or "health utilit*".ti,ab,cl,oa,kw,kf. or "preference elicit*".ti,ab,cl,oa,kw,kf. or "preference score".ti,ab,cl,oa,kw,kf. or "probability trade-off".ti,ab,cl,oa,kw,kf. or "prospect theory".ti,ab,cl,oa,kw,kf. or "time trade-off".ti,ab,cl,oa,kw,kf. or TTO.ti,ab,cl,oa,kw,kf. or best worst scaling.ti,ab,sh,hw,kw,rn,pn,sa,si. or (utility.ti,ab,sh,hw,kw,rn,pn,sa,si. and (value*.ti,ab,sh,hw,kw,rn,pn,sa,si. or score*.ti,ab,sh,hw,kw,rn,pn,sa,si. or estimate*.ti,ab,sh,hw,kw,rn,pn,sa,si.)))  55 (exp Quality of Life/ or "E.Q. 5D".ti,ab,cl,oa,kw,kf. or "EuroQoL 5D".ti,ab,cl,oa,kw,kf. or "multi attribute".ti,ab,cl,oa,kw,kf. or "preference based".ti,ab,cl,oa,kw,kf. or "preference score".ti,ab,cl,oa,kw,kf. or "quality of life".ti,ab,cl,oa,kw,kf. or "S.F. 12".ti,ab,cl,oa,kw,kf. or "S.F. 36".ti,ab,cl,oa,kw,kf. or "S.F. 6D".ti,ab,cl,oa,kw,kf. or 15D.ti,ab,cl,oa,kw,kf. or EQ5D.ti,ab,cl,oa,kw,kf. or EuroQoL5D.ti,ab,cl,oa,kw,kf. or HRQoL.ti,ab,cl,oa,kw,kf. or HUI.ti,ab,cl,oa,kw,kf. or multiattribute.ti,ab,cl,oa,kw,kf. or QoL.ti,ab,cl,oa,kw,kf. or SF12.ti,ab,cl,oa,kw,kf. or SF36.ti,ab,cl,oa,kw,kf. or SF6D.ti,ab,cl,oa,kw,kf.)  56 or/52-55  57 51 and 56  58 limit 57 to yr="2006 - 2022"  59 limit 58 to (english language and humans) |

| ***Values and preferences*** |
| --- |
| **Interface: Embase**  **Database: Embase**  **Type of studies: No restriction** |
| 1 '(('ercp' OR 'endoscopic retrograde cholangiopancreatography/' OR 'endoscop$ retrograd$ cholangiopancreatograph$' OR 'sphincterotomy, endoscopic/' OR 'endoscopic sphincterotom$' OR 'papillotomy') AND ('randomized controlled trial' OR 'controlled clinical trial' OR 'random$' OR trial OR groups) OR ((proton:ti,ab OR nmr:ti,ab OR mr:ti,ab OR magneti*:ti,ab OR computarized:ti,ab OR computarised:ti,ab OR computed:ti,ab) AND (cholangiogra*:ti,ab OR imaging:ti,ab OR scans:ti,ab OR scan:ti,ab OR tomogra*:ti,ab)) OR zeugmatogra*:ti,ab OR nmri:ti,ab OR mri:ti,ab OR tomodensitometry:ti,ab OR ct:ti,ab OR 'cholangiopancreatography, magnetic resonance' OR choledochosco*:ti,ab OR 'cholangiography' OR mrcp:ti,ab OR ercp:ti,ab OR cholledochosco*:ti,ab OR cholangiosco*:ti,ab OR cholangio?pancreatogra*:ti,ab OR cholangiogra*:ti,ab OR 'magnetic resonance imaging' OR 'tomography, x-ray computed') AND (('endoscopy, digestive system/' OR 'cholangioscopy' OR 'cholangiopancreatography, endoscopic retrograde/' OR 'cholangiopancreatoscopy' OR 'cholecystoscopy' OR 'ercp') AND ('single operator' OR 'direct visuali?ation') OR 'spyglass')  2 ('decision making'/exp/mj OR ((‘avoidance behavi*’ OR ‘avoidance learning’ OR decision* OR ‘decision aid*’ OR ‘decision analy*’ OR ‘decision board*’ OR ‘decision mak*’ OR ‘decisions mak*’ OR decision-support OR ‘decision tool*’ OR ‘discrete choice’ OR discrete-choice*):ti,ab) OR ((decision* AND making):ti))  3 ('attitude to health'/exp/mj OR 'patient participation'/exp/mj OR 'patient satisfaction'/exp/mj OR ((choice* OR valuat* OR value*):ti) OR ((acceptab* OR attitude* OR expectation* OR ‘health perception*’ OR ‘health state values’ OR ‘health values’ OR knowledge OR ((patient* OR user*) NEAR/1 (choice* OR participation OR perce* OR perspective* OR valuat* OR value* OR view*)) OR preference*):ti,ab))  4 ('clinical decision support system'/exp/mj OR 'decision support system'/exp/mj OR ((health AND utilit*):ti) OR ((‘best worst’ OR ‘best-worst scaling’ OR ‘feeling thermometer*’ OR gamble* OR ‘health state’ OR ‘health utilit*’ OR ‘preference elicit*’ OR ‘preference score’ OR ‘probability trade-off’ OR ‘prospect theory’ OR ‘time trade-off’ OR TTO):ti,ab ) OR ((‘best worst scaling’ OR (utility AND (value* OR score* OR estimate*))):kw))  5 ('quality of life'/exp/mj OR ((‘E.Q. 5D’ OR ‘EuroQoL 5D’ OR ‘multi attribute’ OR ‘preference based’ OR ‘preference score’ OR ‘quality of life’ OR ‘S.F. 12’ OR ‘S.F. 36’ OR ‘S.F. 6D’ OR 15D OR EQ5D OR EuroQoL5D OR HRQoL OR HUI OR multiattribute OR QoL OR SF12 OR SF36 OR SF6D):ti,ab))  6 (#2 OR #3 OR #4 OR #5)  7 6 AND 1  8 7 AND [english]/lim AND [humans]/lim AND [embase]/lim AND [2006-2022]/py |

| ***Acceptability, equity, feasibility, implementation*** |
| --- |
| **Interface: Ovid**  **Database: Medline**  **Type of studies: No restriction** |
| 1 ERCP.mp.  2 exp endoscopic retrograde cholangiopancreatography/  3 (endoscop$ adj3 retrograd$ adj3 cholangiopancreatograph$).tw.  4 exp Sphincterotomy, Endoscopic/  5 (endoscopic adj3 sphincterotom$).mp.  6 papillotomy.mp.  7 or/1-6  8 CT.ti,ab.  9 tomodensitometry.ti,ab.  10 MRI.ti,ab.  11 NMRI.ti,ab.  12 zeugmatogra*.ti,ab.  13 computed.ti,ab.  14 computerised.ti,ab.  15 computerized.ti,ab.  16 magneti*.ti,ab.  17 MR.ti,ab.  18 NMR.ti,ab.  19 proton.ti,ab.  20 tomogra*.ti,ab.  21 scan.ti,ab.  22 scans.ti,ab.  23 imaging.ti,ab.  24 cholangiogra*.ti,ab.  25 exp Tomography, X-Ray Computed/  26 exp Magnetic Resonance Imaging/  27 cholangiogra*.ti,ab.  28 cholangio?pancreatogra*.ti,ab.  29 cholangiosco*.ti,ab.  30 choledochosco*.ti,ab.  31 ERCP.ti,ab.  32 MRCP.ti,ab.  33 exp Cholangiography/  34 exp Cholangiopancreatography, Magnetic Resonance/  35 8 or 9 or 10 or 11 or 12 or 13 or 14 or 15 or 16 or 17 or 18 or 19 or 20 or 21 or 22 or 23 or 24 or 25 or 26 or 27 or 28 or 29 or 30 or 31 or 32 or 33 or 34  36 Endoscopy, Digestive System/  37 cholangioscopy.mp.  38 Cholangiopancreatography, Endoscopic Retrograde/  39 cholangiopancreatoscopy.mp.  40 cholecystoscopy.mp.  41 ercp.mp.  42 36 or 37 or 38 or 39 or 40 or 41  43 single operator.mp.  44 direct visuali?ation.mp. [mp=title, abstract, original title, name of substance word, subject heading word, floating sub-heading word, keyword heading word, organism supplementary concept word, protocol supplementary concept word, rare disease supplementary concept word, unique identifier, synonyms]  45 43 or 44  46 42 and 45  47 spyglass.mp. [mp=title, abstract, original title, name of substance word, subject heading word, floating sub-heading word, keyword heading word, organism supplementary concept word, protocol supplementary concept word, rare disease supplementary concept word, unique identifier, synonyms]  48 46 or 47  49 remove duplicates from 48  50 7 or 35  51 49 and 50  52 (exp Attitude to Health/ or accepta*.ti,ab,cl,oa,kw,kf. or activat*.ti,ab,cl,oa,kw,kf. or adhere*.ti,ab,cl,oa,kw,kf. or agreement.ti,ab,cl,oa,kw,kf. or attitude*.ti,ab,cl,oa,kw,kf. or belief*.ti,ab,cl,oa,kw,kf. or collaborat*.ti,ab,cl,oa,kw,kf. or complianc*.ti,ab,cl,oa,kw,kf. or comply.ti,ab,cl,oa,kw,kf. or concordan*.ti,ab,cl,oa,kw,kf. or cooperat*.ti,ab,cl,oa,kw,kf. or empower*.ti,ab,cl,oa,kw,kf. or experience*.ti,ab,cl,oa,kw,kf. or inducement.ti,ab,cl,oa,kw,kf. or intent*.ti,ab,cl,oa,kw,kf. or involv*.ti,ab,cl,oa,kw,kf. or motivat*.ti,ab,cl,oa,kw,kf. or negotiat*.ti,ab,cl,oa,kw,kf. or participat*.ti,ab,cl,oa,kw,kf. or partnership.ti,ab,cl,oa,kw,kf. or perception*.ti,ab,cl,oa,kw,kf. or perspective*.ti,ab,cl,oa,kw,kf. or reinforce*.ti,ab,cl,oa,kw,kf. or view*.ti,ab,cl,oa,kw,kf. or willing*.ti,ab,cl,oa,kw,kf.)  53 (exp Cooperative Behavior/ or "patient provider agreement*".ti,ab,cl,oa,kw,kf. or ((shared.ti,ab,cl,oa,kw,kf. or joint.ti,ab,cl,oa,kw,kf. or informed.ti,ab,cl,oa,kw,kf. or collaborative.ti,ab,cl,oa,kw,kf.) and "decision making".ti,ab,cl,oa,kw,kf.) or ((involv*.ti,ab,cl,oa,kw,kf. or participat*.ti,ab,cl,oa,kw,kf.) and (choice*.ti,ab,cl,oa,kw,kf. or decision*.ti,ab,cl,oa,kw,kf.)))  54 52 OR 53  55 (exp *Health Services Accessibility/ or "Access to Health Care".ti,ab,cl,oa,kw,kf. or "Access to Health Service*".ti,ab,cl,oa,kw,kf. or "Access to HealthCare".ti,ab,cl,oa,kw,kf. or "Access To Medic*".ti,ab,cl,oa,kw,kf. or "Access to Medication*".ti,ab,cl,oa,kw,kf. or "Access to Therap*".ti,ab,cl,oa,kw,kf. or "Access to Treat*".ti,ab,cl,oa,kw,kf. or "Accessibility of Health Service*".ti,ab,cl,oa,kw,kf. or "Availability of Health Service*".ti,ab,cl,oa,kw,kf. or coercion.ti,ab,cl,oa,kw,kf. or coercive*.ti,ab,cl,oa,kw,kf. or controvers*.ti,ab,cl,oa,kw,kf. or "Health Services Accessibilit*".ti,ab,cl,oa,kw,kf. or "Health Services Availabilit*".ti,ab,cl,oa,kw,kf. or "Medication Access*".ti,ab,cl,oa,kw,kf. or "Program Accessibilit*".ti,ab,cl,oa,kw,kf.)  56 (exp *Healthcare Disparities/ or equit*.ti,ab,cl,oa,kw,kf. or "Health Care Disparit*".ti,ab,cl,oa,kw,kf. or "Health Care Inequalit*".ti,ab,cl,oa,kw,kf. or "Healthcare Disparit*".ti,ab,cl,oa,kw,kf. or "Healthcare Inequalit*".ti,ab,cl,oa,kw,kf. or inequit*.ti,ab,cl,oa,kw,kf.)  57 (exp *Morals/ or ethics.fs. or ethic*.ti,ab,cl,oa,kw,kf. or fairness.ti,ab,cl,oa,kw,kf. or moral*.ti,ab,cl,oa,kw,kf. or unethical.ti,ab,cl,oa,kw,kf.)  58 or/55-57  59 exp *Feasibility Studies/ or feasib*.ti,ab,cl,oa,kw,kf.  60 effective*.ti,ab,cl,oa,kw,kf. or efficac*.ti,ab,cl,oa,kw,kf.  61 facilita*.ti,ab,cl,oa,kw,kf. or usabilit*.ti,ab,cl,oa,kw,kf.  62 barrier*.ti,ab,cl,oa,kw,kf. or difficult*.ti,ab,cl,oa,kw,kf. or hurdle*.ti,ab,cl,oa,kw,kf. or impede*.ti,ab,cl,oa,kw,kf. or impediment*.ti,ab,cl,oa,kw,kf. or limit*.ti,ab,cl,oa,kw,kf. or obstacle*.ti,ab,cl,oa,kw,kf.  63 or/59-62  64 (exp *Clinical Protocols/ or exp *Consensus/ or exp *Critical Pathways/ or exp Guideline/ or exp *Guidelines as Topic/ or exp *Health Planning Guidelines/ or advice.ti,ab,cl,oa,kw,kf. or advise*.ti,ab,cl,oa,kw,kf. or consensus.ti,ab,cl,oa,kw,kf. or frame-work*.ti,ab,cl,oa,kw,kf. or framework*.ti,ab,cl,oa,kw,kf. or guidance*.ti,ab,cl,oa,kw,kf. or guideline*.ti,ab,cl,oa,kw,kf. or policies.ti,ab,cl,oa,kw,kf. or policy.ti,ab,cl,oa,kw,kf. or protocol*.ti,ab,cl,oa,kw,kf. or recommend*.ti,ab,cl,oa,kw,kf. or standard*.ti,ab,cl,oa,kw,kf. or statement*.ti,ab,cl,oa,kw,kf.)  65 (accordance.ti,ab,cl,oa,kw,kf. or adhere*.ti,ab,cl,oa,kw,kf. or adopt*.ti,ab,cl,oa,kw,kf. or aware*.ti,ab,cl,oa,kw,kf. or barrier*.ti,ab,cl,oa,kw,kf. or compliance*.ti,ab,cl,oa,kw,kf. or complies.ti,ab,cl,oa,kw,kf. or comply*.ti,ab,cl,oa,kw,kf. or concordance.ti,ab,cl,oa,kw,kf. or disseminat*.ti,ab,cl,oa,kw,kf. or facilitat*.ti,ab,cl,oa,kw,kf. or implement*.ti,ab,cl,oa,kw,kf. or incorporat*.ti,ab,cl,oa,kw,kf. or integrat*.ti,ab,cl,oa,kw,kf. or spread*.ti,ab,cl,oa,kw,kf. or sustain*.ti,ab,cl,oa,kw,kf. or takeup*.ti,ab,cl,oa,kw,kf. or take-up*.ti,ab,cl,oa,kw,kf. or uptake*.ti,ab,cl,oa,kw,kf. or up-take*.ti,ab,cl,oa,kw,kf.)  66 64 and 65  67 (exp *Diffusion of Innovation/ or exp *Health Plan Implementation/ or exp *Information Dissemination/ or exp *Guideline Adherence/ or exp *Organizational Innovation/ or "Guideline Implementation".ti,ab,cl,oa,kw,kf. or "Health Plan Implementation*".ti,ab,cl,oa,kw,kf. or "Information Dissemination".ti,ab,cl,oa,kw,kf. or "Information Distribution".ti,ab,cl,oa,kw,kf. or "Innovation Diffusion".ti,ab,cl,oa,kw,kf. or "Institutional Implementation".ti,ab,cl,oa,kw,kf. or "Policy Implementation".ti,ab,cl,oa,kw,kf. or "Protocol Implementation".ti,ab,cl,oa,kw,kf.)  68 66 or 67  69 54 or 58 or 63 or 68  70 51 and 69  71 limit to yr="2006 - 2022"  72 limit to (english language and humans) |

| ***Acceptability*** |
| --- |
| **Interface: Embase**  **Database: Embase**  **Contextual Factor: Acceptability**  **Type of studies: No restriction** |
| 1 '(('ercp' OR 'endoscopic retrograde cholangiopancreatography/' OR 'endoscop$ retrograd$ cholangiopancreatograph$' OR 'sphincterotomy, endoscopic/' OR 'endoscopic sphincterotom$' OR 'papillotomy') AND ('randomized controlled trial' OR 'controlled clinical trial' OR 'random$' OR trial OR groups) OR ((proton:ti,ab OR nmr:ti,ab OR mr:ti,ab OR magneti*:ti,ab OR computarized:ti,ab OR computarised:ti,ab OR computed:ti,ab) AND (cholangiogra*:ti,ab OR imaging:ti,ab OR scans:ti,ab OR scan:ti,ab OR tomogra*:ti,ab)) OR zeugmatogra*:ti,ab OR nmri:ti,ab OR mri:ti,ab OR tomodensitometry:ti,ab OR ct:ti,ab OR 'cholangiopancreatography, magnetic resonance' OR choledochosco*:ti,ab OR 'cholangiography' OR mrcp:ti,ab OR ercp:ti,ab OR cholledochosco*:ti,ab OR cholangiosco*:ti,ab OR cholangio?pancreatogra*:ti,ab OR cholangiogra*:ti,ab OR 'magnetic resonance imaging' OR 'tomography, x-ray computed') AND (('endoscopy, digestive system/' OR 'cholangioscopy' OR 'cholangiopancreatography, endoscopic retrograde/' OR 'cholangiopancreatoscopy' OR 'cholecystoscopy' OR 'ercp') AND ('single operator' OR 'direct visuali?ation') OR 'spyglass')  2 ('attitude to health'/exp OR ((accepta* OR activat* OR adhere* OR agreement OR attitude* OR belief* OR collaborat* OR complianc* OR comply OR concordan* OR cooperat* OR co‐operat* OR empower* OR experience* OR inducement OR intent* OR involv* OR motivat* OR negotiat* OR participat* OR partnership OR perception* OR perspective* OR reinforce* OR view* OR willing*):ti,ab))  3 ('cooperation'/exp OR ("patient provider agreement*" OR ((shared OR joint OR informed OR collaborative) AND "decision making") OR ((involv* OR participat*) AND (choice* OR decision*):ti,ab)))  4 (#2 OR #3)  5 4 AND 1  6 '5 AND [english]/lim AND [humans]/lim AND [embase]/lim AND [2006-2022]/py |

| ***Equity*** |
| --- |
| **Interface: Embase**  **Database: Embase**  **Type of studies: No restriction** |
| 1 '(('ercp' OR 'endoscopic retrograde cholangiopancreatography/' OR 'endoscop$ retrograd$ cholangiopancreatograph$' OR 'sphincterotomy, endoscopic/' OR 'endoscopic sphincterotom$' OR 'papillotomy') AND ('randomized controlled trial' OR 'controlled clinical trial' OR 'random$' OR trial OR groups) OR ((proton:ti,ab OR nmr:ti,ab OR mr:ti,ab OR magneti*:ti,ab OR computarized:ti,ab OR computarised:ti,ab OR computed:ti,ab) AND (cholangiogra*:ti,ab OR imaging:ti,ab OR scans:ti,ab OR scan:ti,ab OR tomogra*:ti,ab)) OR zeugmatogra*:ti,ab OR nmri:ti,ab OR mri:ti,ab OR tomodensitometry:ti,ab OR ct:ti,ab OR 'cholangiopancreatography, magnetic resonance' OR choledochosco*:ti,ab OR 'cholangiography' OR mrcp:ti,ab OR ercp:ti,ab OR cholledochosco*:ti,ab OR cholangiosco*:ti,ab OR cholangio?pancreatogra*:ti,ab OR cholangiogra*:ti,ab OR 'magnetic resonance imaging' OR 'tomography, x-ray computed') AND (('endoscopy, digestive system/' OR 'cholangioscopy' OR 'cholangiopancreatography, endoscopic retrograde/' OR 'cholangiopancreatoscopy' OR 'cholecystoscopy' OR 'ercp') AND ('single operator' OR 'direct visuali?ation') OR 'spyglass')  2 ('health care access'/exp/mj OR (("Access to Health Care" OR "Access to Health Service*" OR "Access to HealthCare" OR "Access To Medic*" OR "Access to Medication*" OR "Access to Therap*" OR "Access to Treat*" OR "Accessibility of Health Service*" OR "Availability of Health Service*" OR coercion OR coercive* OR controvers* OR "Health Services Accessibilit*" OR "Health Services Availabilit*" OR "Medication Access*" OR "Program Accessibilit*"):ti,ab))  3 ('health care disparity'/exp/mj OR ((equit* OR "Health Care Disparit*" OR "Health Care Inequalit*" OR "Healthcare Disparit*" OR "Healthcare Inequalit*" OR inequit*):ti,ab))  4 ('morality'/exp/mj OR 'ethics'/exp/mj OR ((ethic* OR equity OR moral* OR unethical):ti,ab))  5 (#2 OR #3 OR #4)  6 5 AND 1  7 6 AND [english]/lim AND [humans]/lim AND [embase]/lim AND [2006-2022]/py |

| ***Feasibility*** |
| --- |
| **Interface: Embase**  **Database: Embase**  **Type of studies: No restriction** |
| 1 '(('ercp' OR 'endoscopic retrograde cholangiopancreatography/' OR 'endoscop$ retrograd$ cholangiopancreatograph$' OR 'sphincterotomy, endoscopic/' OR 'endoscopic sphincterotom$' OR 'papillotomy') AND ('randomized controlled trial' OR 'controlled clinical trial' OR 'random$' OR trial OR groups) OR ((proton:ti,ab OR nmr:ti,ab OR mr:ti,ab OR magneti*:ti,ab OR computarized:ti,ab OR computarised:ti,ab OR computed:ti,ab) AND (cholangiogra*:ti,ab OR imaging:ti,ab OR scans:ti,ab OR scan:ti,ab OR tomogra*:ti,ab)) OR zeugmatogra*:ti,ab OR nmri:ti,ab OR mri:ti,ab OR tomodensitometry:ti,ab OR ct:ti,ab OR 'cholangiopancreatography, magnetic resonance' OR choledochosco*:ti,ab OR 'cholangiography' OR mrcp:ti,ab OR ercp:ti,ab OR cholledochosco*:ti,ab OR cholangiosco*:ti,ab OR cholangio?pancreatogra*:ti,ab OR cholangiogra*:ti,ab OR 'magnetic resonance imaging' OR 'tomography, x-ray computed') AND (('endoscopy, digestive system/' OR 'cholangioscopy' OR 'cholangiopancreatography, endoscopic retrograde/' OR 'cholangiopancreatoscopy' OR 'cholecystoscopy' OR 'ercp') AND ('single operator' OR 'direct visuali?ation') OR 'spyglass')  2 ('feasibility study'/exp/mj OR ((feasib*):ti,ab))  3 ((effective* OR efficac*):ti,ab)  4 ((facilita* OR usabilit*):ti,ab)  5 ((barrier* OR difficult* OR hurdle* OR impede* OR impediment* OR limit* OR obstacle*):ti,ab)  6 (#2 OR #3 OR #4 OR #5)  7 6 AND 1  8 7 AND [english]/lim AND [humans]/lim AND [embase]/lim AND [2006-2022]/py |

| ***Implementation*** |
| --- |
| **Interface: Embase**  **Database: Embase**  **Type of studies: No restriction** |
| 1 '(('ercp' OR 'endoscopic retrograde cholangiopancreatography/' OR 'endoscop$ retrograd$ cholangiopancreatograph$' OR 'sphincterotomy, endoscopic/' OR 'endoscopic sphincterotom$' OR 'papillotomy') AND ('randomized controlled trial' OR 'controlled clinical trial' OR 'random$' OR trial OR groups) OR ((proton:ti,ab OR nmr:ti,ab OR mr:ti,ab OR magneti*:ti,ab OR computarized:ti,ab OR computarised:ti,ab OR computed:ti,ab) AND (cholangiogra*:ti,ab OR imaging:ti,ab OR scans:ti,ab OR scan:ti,ab OR tomogra*:ti,ab)) OR zeugmatogra*:ti,ab OR nmri:ti,ab OR mri:ti,ab OR tomodensitometry:ti,ab OR ct:ti,ab OR 'cholangiopancreatography, magnetic resonance' OR choledochosco*:ti,ab OR 'cholangiography' OR mrcp:ti,ab OR ercp:ti,ab OR cholledochosco*:ti,ab OR cholangiosco*:ti,ab OR cholangio?pancreatogra*:ti,ab OR cholangiogra*:ti,ab OR 'magnetic resonance imaging' OR 'tomography, x-ray computed') AND (('endoscopy, digestive system/' OR 'cholangioscopy' OR 'cholangiopancreatography, endoscopic retrograde/' OR 'cholangiopancreatoscopy' OR 'cholecystoscopy' OR 'ercp') AND ('single operator' OR 'direct visuali?ation') OR 'spyglass')  2 ('clinical protocol'/exp/mj OR 'consensus'/exp/mj OR 'clinical pathway'/exp/mj OR 'guideline'/exp/mj OR 'health care planning'/exp/mj OR ((advice OR advise* OR consensus OR framework* OR framework* OR guidance* OR guideline* OR policies OR policy OR protocol* OR recommend* OR standard* OR statement*):ti,ab))  3 ((accordance OR adhere* OR adopt* OR aware* OR barrier* OR compliance* OR complies OR comply* OR concordance OR disseminat* OR facilitat* OR implement* OR incorporat* OR integrat* OR spread* OR sustain* OR takeup* OR take-up* OR uptake* OR up-take*):ti,ab)  4 2 AND 3  5 ('diffusion of innovation'/exp/mj OR 'health care planning'/exp/mj OR 'information dissemination'/exp/mj OR 'protocol compliance'/exp/mj OR 'organization'/exp/mj OR (("Guideline Implementation" OR "Health Plan Implementation*" OR "Information Dissemination" OR "Information Distribution" OR "Innovation Diffusion" OR "Institutional Implementation" OR "Policy Implementation" OR "Protocol Implementation"):ti,ab))  6 (#4 OR #5)  7 6 AND 1  8 7 AND [english]/lim AND [humans]/lim AND [embase]/lim AND [2006-2022]/py |
